# Spatial disparity and factors associated with dementia mortality: A cross-sectional study in Zhejiang Province, China

**DOI:** 10.3389/fpubh.2023.1100960

**Published:** 2023-03-24

**Authors:** Xiaotian Heng, Xiaoting Liu, Na Li, Jie Lin, Xiaoyan Zhou

**Affiliations:** ^1^School of Public Health, Zhejiang University, Hangzhou, China; ^2^School of Public Affairs, Zhejiang University, Hangzhou, China; ^3^Department of Chronic Disease Prevention and Control, Zhejiang Provincial Center for Disease Control and Prevention, Hangzhou, China

**Keywords:** dementia mortality, death surveillance, spatial analysis, disease mapping, BYM modeling

## Abstract

**Objective:**

Evidence of spatial disparity in dementia mortality in China has been found to have higher dementia mortality in eastern and rural China. Regional factors of physical and social features may be influencing this spatial disparity. However, the extent of spatial difference in dementia mortality across small regional localities is unclear. This study aims to investigate the geographic variations in mortality and risk of all dementia subtypes and identify the effect of the associated environmental risk factors.

**Methods:**

We used surveillance data on death reports from Alzheimer’s disease and other forms of dementia in Zhejiang province from 2015 to 2019. We estimated the relative risk of dementia mortality using a Bayesian spatial model. We mapped predicted relative risk to visualize the risk of death from different types of dementia and to identify risk factors associated with dementia.

**Results:**

Thirty thousand three hundred and ninety-eight deaths attributable to dementia as the underlying or related cause (multiple causes) were reported during 2015–2019. Counties and districts in the southeast and west of Zhejiang province had significantly higher standardized mortality ratios than others. Counties and districts with a smaller proportion of residents aged 60 years or older, poorer economic status, insufficient health resources, and worse pollution had a higher risk of deaths due to dementia.

**Conclusion:**

Higher risks of dementia mortality were found in counties and districts with poorer economic status, insufficient health resources, and worse pollution in Zhejiang. Our study adds new evidence on the association between socioeconomic and environmental factors and the mortality risk due to dementia.

## 1. Introduction

Dementia is a significant public health challenge in China and other low- and middle-income countries. Around 55 million people have dementia worldwide, with nearly 60% living in low- and middle-income countries (1). Dementia, including Alzheimer’s disease (AD) and other forms of dementia, is one of the major causes of disability and death among older people and is now among the top 10 causes of death overall (2). In the United States, AD is officially ranked as the sixth leading cause of death and the fifth leading cause of death for people aged 65 and over (3). There is often a lack of awareness and understanding of dementia among older adults, resulting in barriers to dementia diagnosis and care, which may cause even higher death numbers than the official sources acknowledged.

Few studies have investigated spatial disparity in mortality from dementia in China (4–6). One study used mortality data from China National Disease Surveillance system and reported dementia mortality is significantly higher in eastern China than in the western and central regions, with a rate ratio of 2.28 (95% CI 1.45–3.60) (4). The risk of dementia is higher in rural areas than in urban areas (6). Some multicenter surveys have also shown that dementia prevalence is higher in rural China than in urban areas (7, 8). These surveys did not examine the relationships between the risk of dementia and social characteristics in small areas, and the specific explanation for the spatial disparity in dementia mortality risk is not known yet. Risk factors may be spatially correlated because individuals in the same region tend to have similar characteristics. Analyzes of risk factors for dementia have rarely considered the spatial correlation of disease. One measure of the risk of death from disease is the standardized mortality ratio. However, in many cases, small areas may have extremely standardized mortality ratios due to small population sizes or small samples. A Bayesian spatial model is preferred in this case to estimate the disease risk in small areas. It can borrow information from neighboring areas and incorporate covariate information so that extreme values are smoothed or shrunk (9). Zhejiang, a province in southeast China, has a population of 11 million people aged 60 or over. A cross-sectional survey showed that the age-standardized prevalence of dementia, AD, and vascular dementia in Zhejiang province was 13.0, 6.9, and 0.5%, respectively (10).

Several studies have indicated that dementia mortality varies spatially across different developed countries (11, 12). Cross-national comparisons of dementia death data (ICD-10: F01, F02, F03, and G30) from European and non-European countries revealed that the United States, Canada, France, Spain, and Nigeria had higher proportions of deaths attributable to dementia than Hungary, Mexico, and South Korea (13–15). A study on the spatial distribution of deaths due to Alzheimer’s disease in São Paulo state, Brazil, found that areas with higher death mortality due to Alzheimer’s disease were concentrated in the northern and northwestern parts of the state (16). The spatial disparity in dementia mortality among countries or regions may result from various factors, such as biological, environmental, and social determinants.

This study used data from dementia death certificates in Zhejiang province from 2015 to 2019 provided by the disease surveillance information reporting management system of the Chinese Center for Disease Prevention. It aims to explore the geographical variations in dementia mortality, estimate the spatial risk of deaths due to dementia using Bayesian spatial models, and identify the effect of the associated environmental and socioeconomic risk factors. We also mapped the predicted relative risk to visualize and identify districts at high risk for intervention and further study.

## 2. Methods

### 2.1. Data source and study population

The mortality data presented in this study was collected from the 2015–2019 Chronic Disease Surveillance Information and Management System provided by the Zhejiang Provincial Center for Disease Control and Prevention. This organization has gathered death certificates from all counties and districts in Zhejiang province since 2015. Zhejiang has had 90 counties and districts since the end of 2019, so the data for this study included 89 counties and districts. The death certificates contain medical information, such as causes of death from the immediate cause to underlying causes and other comorbidities or morbid states. Cause-of-death codes have been classified in terms of the International Classification of Diseases, 10th Revision (ICD–10) since 2000. After physicians upload data to the system, specialists analyze the diagnoses declared on the death cases to ensure data accuracy. Crude mortality rates are calculated based on the population statistical bulletin of Zhejiang published by the Statistical Bureau.

AD is the most common cause of dementia, accounting for an estimated 60 to 80% of cases (17). Other major forms of dementia include vascular dementia, dementia with Lewy bodies, and a group of diseases that contribute to frontotemporal dementia. The lines between the different types of dementia are blurred, and mixed forms often coexist. Four categories of dementia were included in this study which adopted international definitions of AD and other dementia: vascular dementia (F01.0, F01.1, F01.2, F01.3, F01.8, and F01.9); unspecified dementia (F03); Alzheimer’s disease (G30.0, G30.1, G30.8, G30.9); and other degenerative diseases of the nervous system not elsewhere classified (G31), including Lewy body disease and frontotemporal dementia (18). All the causes of death (both underlying causes and associated causes) mentioned in these four categories of dementia on the death certificates were included during the selection since the underlying death of a person with dementia may be caused by other conditions. WHO defines an underlying cause as the “disease or injury which initiated the train of events leading directly to death, or the circumstances of the accident or violence which produced the fatal injury.”

### 2.2. Covariates

Previous evidence showed dementia death rates associated with metal elements in drinking water, PM2.5, environmental tobacco smoke, and elements in soil (19–21). The variable indicators for counties and districts from 2015 to 2019 used in this study were obtained from the Zhejiang Statistical Yearbook and included the proportion of people aged 60 years or older, GDP *per capita*, healthcare resource indicators, and PM2.5 counts.

### 2.3. Statistical analysis

We first conducted descriptive statistics of death cases from different types of dementia. We calculated standardized mortality ratios of all dementia subtypes each year from 2015 to 2019, and calculated standardized mortality ratios of vascular dementia, unspecified dementia, Alzheimer’s disease, and other degenerative diseases of the nervous system not elsewhere classified in 5 years total. Then, we used a Bayesian spatial method to get relative risk estimates of dementia mortality in 89 counties in Zhejiang province and map standardized mortality ratio and relative risk of dementia mortality. We also investigated the effect of risk factors associated with dementia mortality. The data used in the analysis contained all dementia deaths.

We fit the Besag-York-Mollié (BYM) model to estimate relative risks in counties. The BYM model is a popular Bayesian spatial model and the most used disease mapping method, named after its authors Besag et al. (22). It is a convolution of a spatially structured random effect that smoothes the data according to a neighborhood structure and a spatially unstructured exchangeable component that models uncorrelated noise (23). Disease mapping is commonly used in small areas to assess a particular disease’s pattern, highlight regions with high relative risk, and identify risk factors. BYM model for disease mapping is a flexible and robust method that accounts for the effects of covariates, incorporates spatial and spatial correlation, and expresses uncertainty in the risk estimates (24, 25). Bayesian approaches use probability to measure uncertainty in predictions or inference estimates and incorporate spatial and temporal dependencies by specifying prior distributions.

Using the notation of Moraga (9), the BYM model is as follows:


$$Y_{i}\sim \text{Poisson}\left(E_{i}\theta_{i}\right),i=1,\cdots ,n,$$

$$\log\left(\theta_{i}\right)=\beta_{0}+d{\left(x_{i}\right)}^{\prime }\beta +u_{i}+v_{i},$$

The outcome variable is the number of dementia death cases per county. Dementia deaths are generally considered to follow a Poisson distribution. We specify *Y_i_* to be the counts of dementia death cases in county *i*. We assumed that *Y_i_* is conditionally independently Poisson distributed, *E_i_* is the expected number of dementia death cases in county *i*, *θ_i_* is the relative risk in county *i*, and *n* is the number of counties. We expressed *β_0_* is the intercept parameter that represents the overall risk, *d(.)* is a vector of observed covariates, *β* is the coefficients of the covariates, *u_i_* is a spatial structured effect component modeled with a conditional auto-regressive (CAR) distribution as $u_{i}|u_{-i}∼N({\bar{u}}_{\delta_{i}},\frac{\sigma_{u}^{2}}{n\delta_{i}})$, and *v_i_* is an unstructured spatial effect defined as $v_{i}\sim N\left(0,\sigma_{v}^{2}\right)$. The relative risk *θ_i_* quantifies whether county *i* has a higher (*θ_i_* > 1) or lower (*θ_i_* < 1) risk than the average risk in the standard population. We produced the probabilities of predicted relative risk being greater than a given threshold *c* [exceedance probabilities, i.e., *P* (*θ_i_* > *c*)]. We implemented the Poisson model by the recommended strategy [i.e., integrating the nested Laplace approximation (INLA)]. Due to the lack of reliable *a priori* information on all parameters, we used a non-informative approach to select the prior and used the default available in the R-INLA package. All analyzes were performed using the R-INLA package (26–28). This study was conducted following the Declaration of Helsinki. The informed consents were obtained through death cases collected by the Center for Disease Control and Prevention (CDC) of Zhejiang Province. The Ethics Committee of Zhejiang CDC approved this study.

## 3. Results

### 3.1. Death characteristics

**Figure 1:**
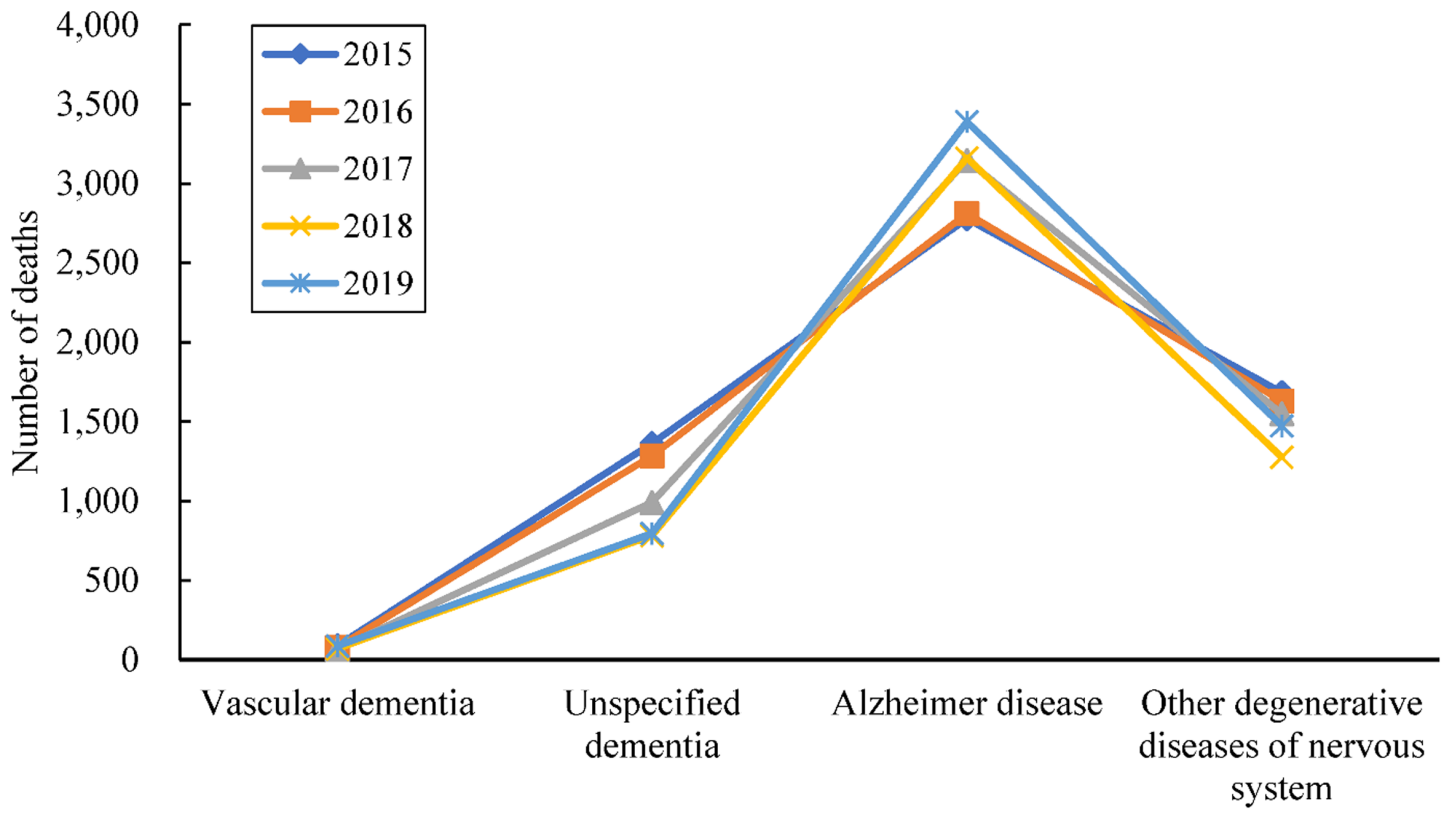
The number of deaths attributed to four dementia subtypes as an underlying cause of death in Zhejiang, 2015–2019.

**Figure 2:**
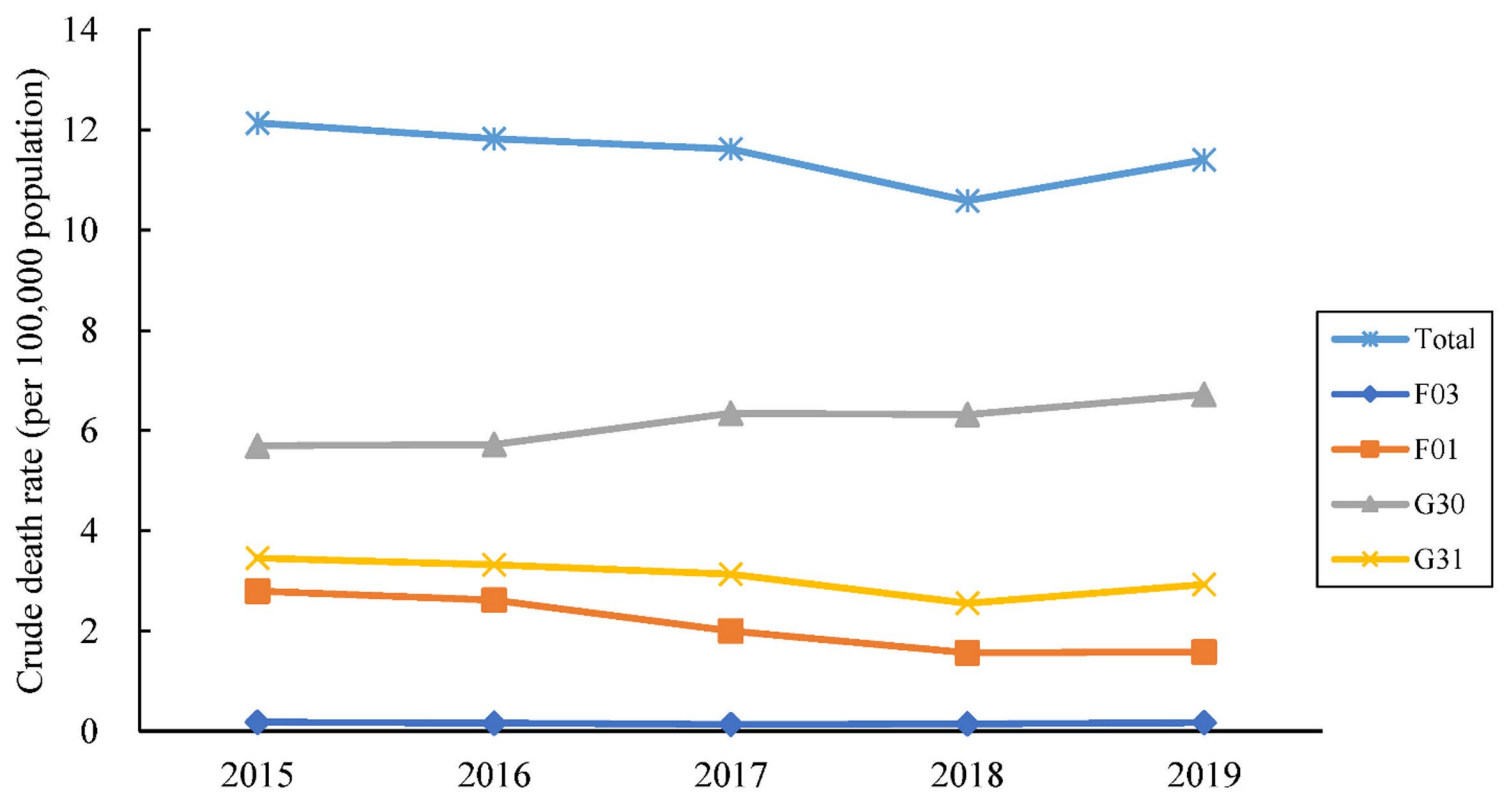
The crude death rates (per 100,000 population) attributed to four dementia subtypes as an underlying cause of death in Zhejiang, 2015–2019. Dementia deaths are identified according to the International Classification of Diseases, 10th Revision underlying cause-of-death codes: F03 (unspecified dementia), G30 (Alzheimer disease), F01 (vascular dementia), and G31 (other degenerative diseases of nervous system).

Death certificates mentioned AD or other dementia as the underlying cause or a related cause (multiple causes) accounted for 30,398 during 2015–2019 in Zhejiang. Of the 28,513 deaths (93.80%) with AD or dementia as the underlying cause, 54% were caused by AD (Figure 1). Figure 2 shows that the temporal trend of mortality between the four dementia subtypes varied. The mortality of Alzheimer’s disease shows an upward trend, while the mortality of unspecified dementia exhibits a downward trend. 57.9 % of deaths due to dementia are women (Table 1). The mean age at the time of death was 83.3 years (maximum 112 years), with age marginally higher in women by 2.23 years than for men. Most males were married (i.e., wife still alive) at the time of death (63.6% for males and 35.7% for females), while females were usually widowed (63.0% for females and 31.0% for males). The place of death of people with dementia was at home in most cases.

**Table 1:** Characteristics of death cases attributable to dementia during 2015–2019 in Zhejiang.

	Overall	Males	Females
	*n* = 30,398	*n* = 12,800 (42.1%)	*n* = 17,598 (57.9%)
Mean age	83.3	82.0	84.2
Standard deviation	9.03	9.47	8.57
Marital status			
Single	658 (2.16%)	541 (4.23%)	117 (0.66%)
Married	14,414 (47.4%)	8,138 (63.6%)	6,276 (35.7%)
Widow(er)	15,052 (49.5%)	3,968 (31.0%)	11,084 (63.0%)
Divorced	212 (0.70%)	119 (0.93%)	93 (0.53%)
Unspecified	62 (0.20%)	34 (0.27%)	28 (0.16%)
Education degree			
Postgraduate	9 (0.03%)	5 (0.04%)	4 (0.02%)
University	165 (0.54%)	119 (0.93%)	46 (0.26%)
Junior college	110 (0.36%)	64 (0.50%)	46 (0.26%)
Secondary school	161 (0.53%)	80 (0.62%)	81 (0.46%)
Technical school	6 (0.02%)	4 (0.03%)	2 (0.01%)
High school	466 (1.53%)	271 (2.12%)	195 (1.11%)
Below	29,481 (97.0%)	12,257 (95.8%)	17,224 (97.9%)
Place of death			
Unspecified	16 (0.05%)	8 (0.06%)	8 (0.05%)
Medical institution	1,763 (5.80%)	868 (6.78%)	895 (5.09%)
The way to the hospital	127 (0.42%)	64 (0.50%)	63 (0.36%)
Home	27,495 (90.5%)	11,383 (88.9%)	16,112 (91.6%)
Retirement home	778 (2.56%)	351 (2.74%)	427 (2.43%)
Other sites	219 (0.72%)	126 (0.98%)	93 (0.53%)
Age			
<60	627 (2.06%)	354 (2.77%)	273 (1.55%)
60–64	574 (1.89%)	310 (2.42%)	264 (1.50%)
65–69	1,025 (3.37%)	544 (4.25%)	481 (2.73%)
70–74	1,725 (5.67%)	877 (6.85%)	848 (4.82%)
75–79	3,379 (11.1%)	1,621 (12.7%)	1,758 (9.99%)
80–84	7,427 (24.4%)	3,289 (25.7%)	4,138 (23.5%)
85–89	8,798 (28.9%)	3,511 (27.4%)	5,287 (30.0%)
90–94	5,301 (17.4%)	1,848 (14.4%)	3,453 (19.6%)
95–99	1,377 (4.53%)	402 (3.14%)	975 (5.54%)
100+	165 (0.54%)	44 (0.34%)	121 (0.69%)

### 3.2. Mapping standardized mortality ratio and relative risk of dementia mortality in Zhejiang

**Figure 3:**
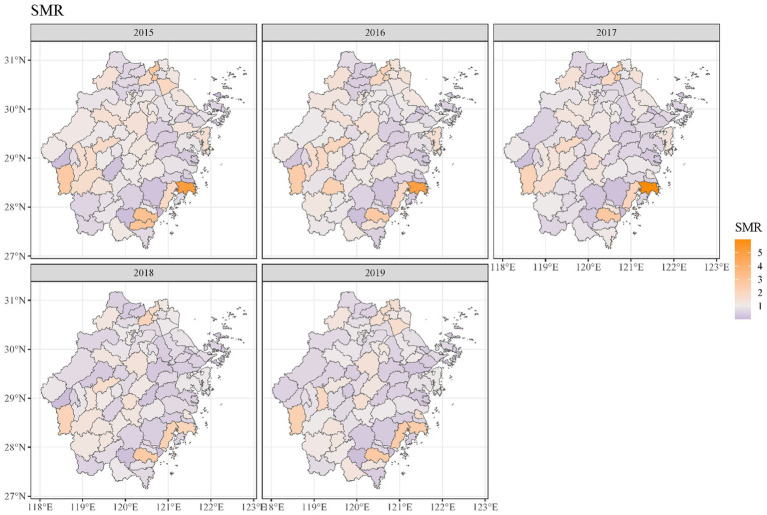
Maps of dementia standardized mortality ratio in Zhejiang counties from 2015 to 2019.

**Figure 4:**
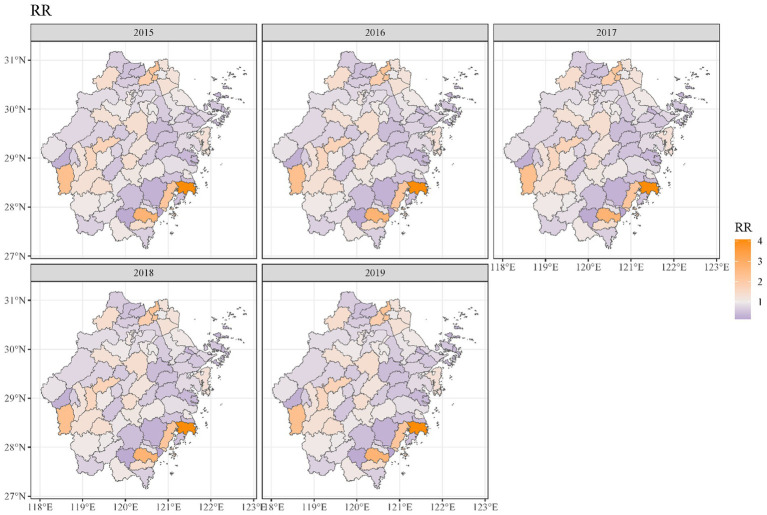
Maps of dementia relative risk in Zhejiang counties from 2015 to 2019.

**Figure 5:**
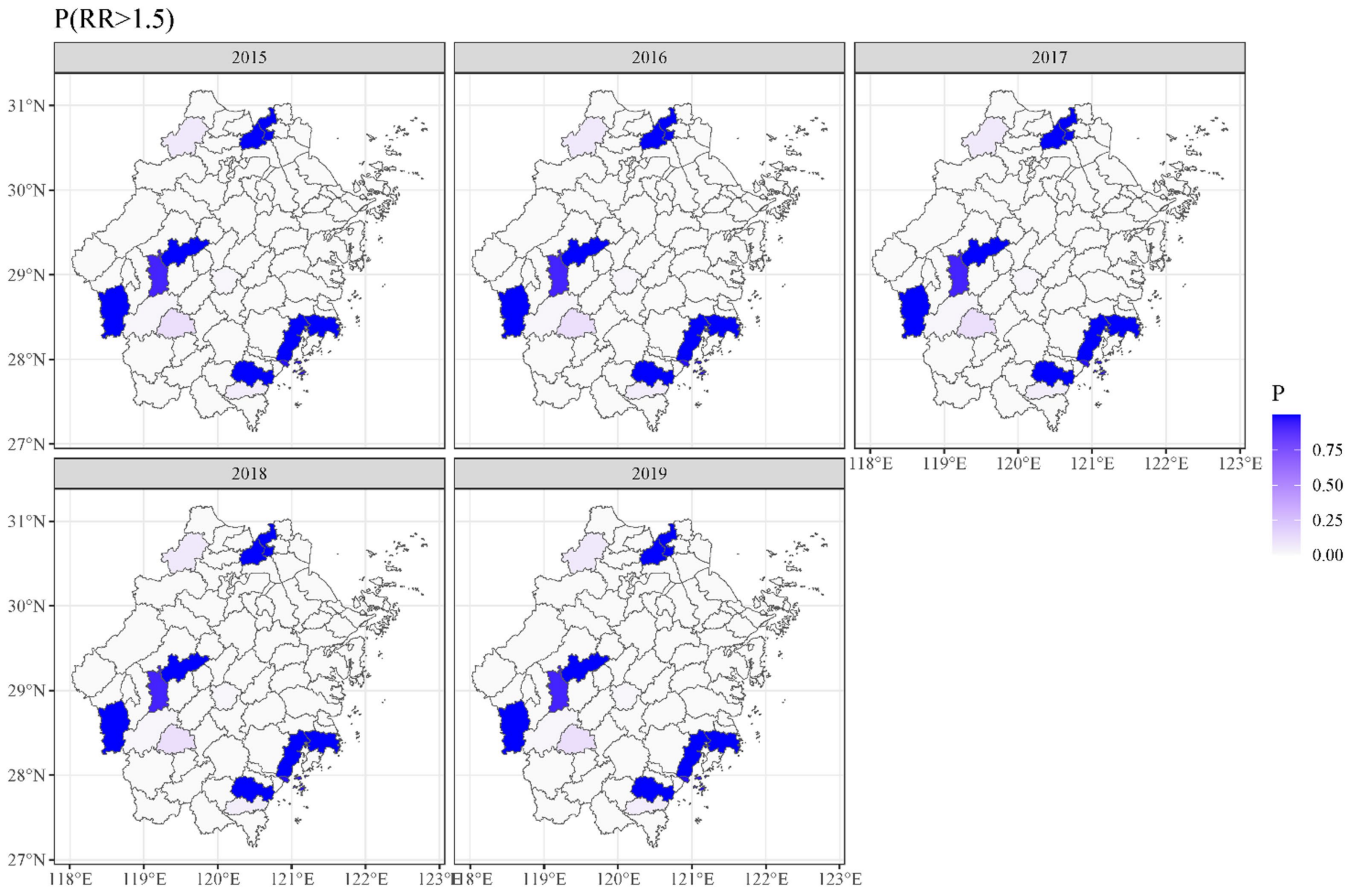
Maps of dementia predicted relative risk exceeding 1.5.

**Figure 6:**
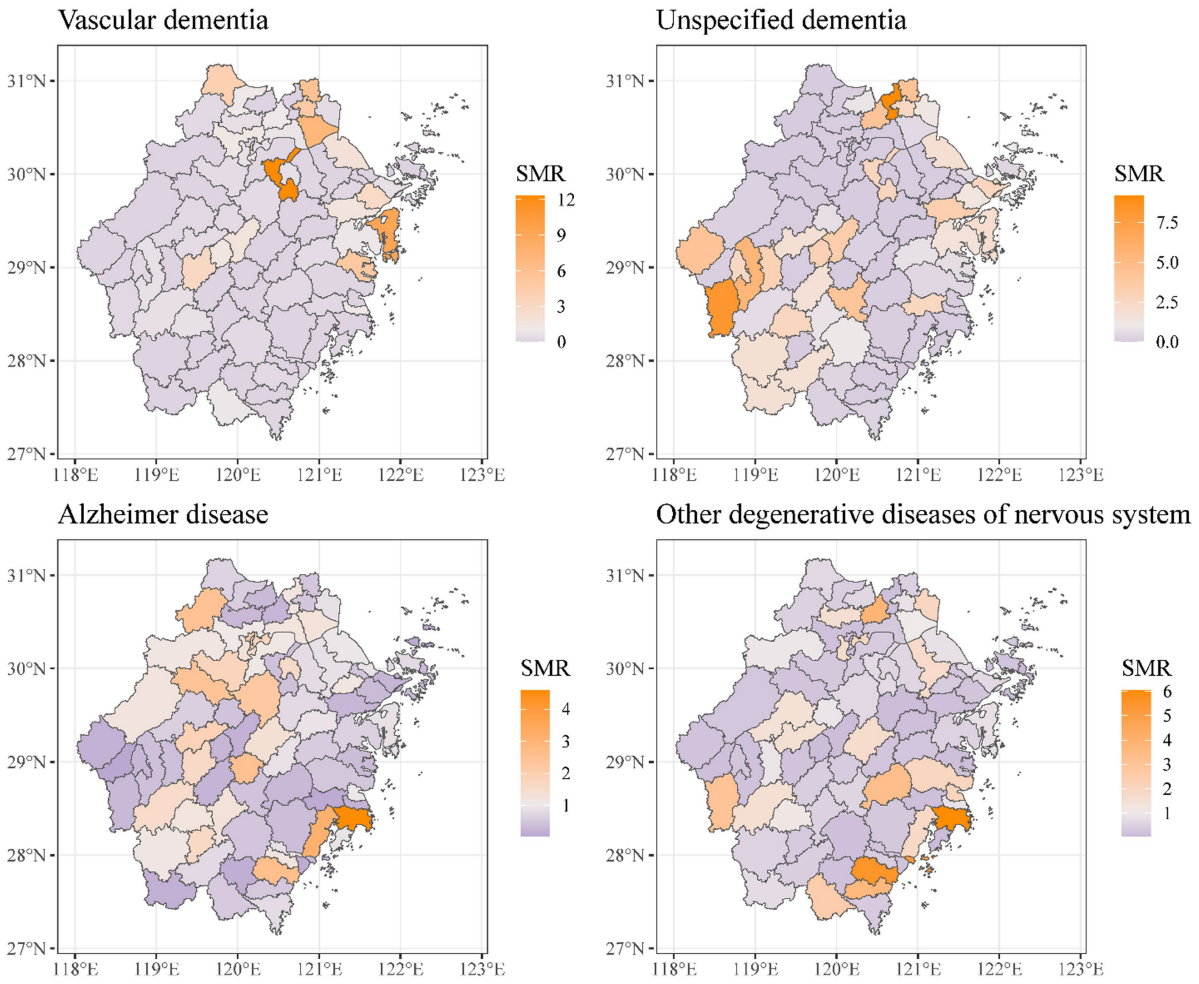
Maps of standardized mortality ratio in Zhejiang counties for four dementia subtypes as an underlying cause of death.

Geographical variations in dementia mortality were found in the study (mortality of all types of dementia). As seen in Figure 3, the standardized mortality ratios of counties and districts in south-eastern and western Zhejiang province were higher than in other counties and districts. We found that the risk of dementia mortality was significantly high among older adults living in the southeast of Zhejiang province (Figure 4). The predicted mean relative risk was 0.98, ranging from 0.14 to 4.37. Figure 5 presents the predicted relative risk exceeding 1.5 for counties in Zhejiang. Figure 6 illustrates the distinct distributions of geographical variations in dementia mortality for four dementia subtypes. The standardized mortality ratios for vascular dementia and unspecified dementia were higher in counties in the north of Zhejiang, which are inconsistent with the distributions of mortality of Alzheimer disease and other degenerative diseases of the nervous system.

### 3.3. Risk factors of dementia mortality from the spatial model

**Figure 7:**
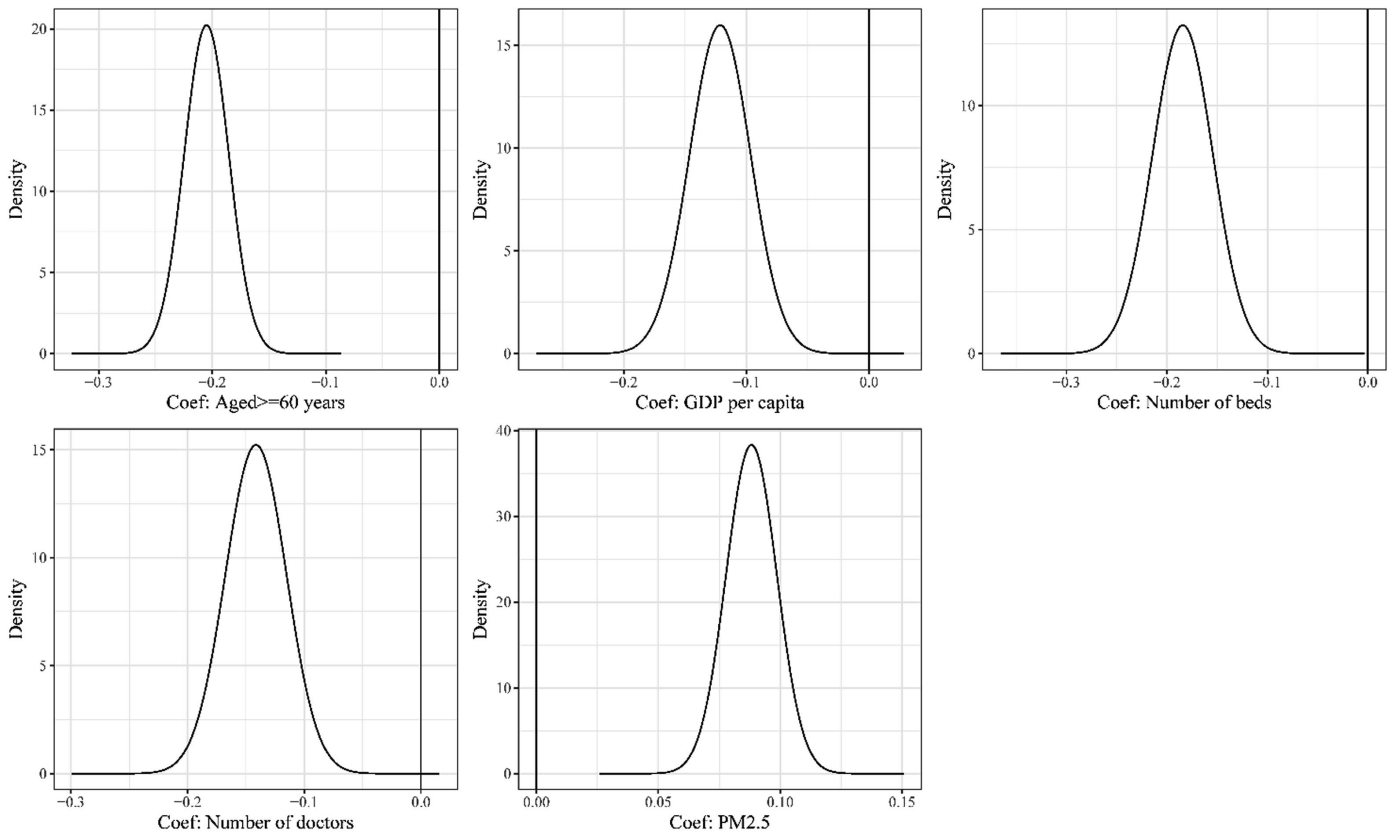
Risk factors associated with dementia mortality (Coef: Coefficient).

Table 2 shows the distributions of the characteristics of socioeconomic factors and environments in the 89 counties and districts in Zhejiang province. In the Bayesian spatial model, the proportion of residents aged above 60 years, GDP *per capita*, number of hospital beds, number of doctors, and counts of PM2.5 were risk factors independently associated with the risk of deaths due to dementia. Counties and districts with poorer economics, insufficient health resources, and worse pollution are associated with a higher risk of dementia mortality (Figure 7).

**Table 2:** Descriptive statistics of risk factors for counties and districts (*n* = 89) in Zhejiang.

Factors	Min	Median	Max	Mean ± SD
People aged ≥60 (%)	13.47	21.75	34.29	21.94 ± 3.59
GDP *per capita*	18,053	83,598	375,963	91,894 ± 49460.61
Number of hospital beds	204	2,750	14,205	3,559 ± 2831.40
Number of doctors	181	1748	8,842	2,292 ± 1710.80
PM2.5 counts	14.88	31.33	66.38	33.66 ± 10.55

## 4. Discussion

This cross-sectional study used a Bayesian spatial method to analyze the spatial distribution characteristics of dementia mortality in Zhejiang from 2015 to 2019 and to evaluate factors associated with a high death rate of dementia. Our results found the spatial disparity for mortality of all dementia subtypes in Zhejiang: districts with the highest risk were in the southeast and west. We have discussed several aspects of social and economic conditions related to dementia mortality and after further investigation of high-risk areas, we revealed that these counties and districts shared similar circumstances, usually with worse economics, insufficient local health resources, and more polluted environments. Such spatial variations of dementia mortality may be attributed to the commons of neighboring regions, such as the geographical context, socioeconomic backgrounds, availability of medical services, and the similarity of residents’ modes of living, but the mechanisms by which these characteristics affect dementia mortality in small areas are currently unclear. Spatial heterogeneity in dementia mortality exhibits across countries and regions due to the influence of multiple risk factors, such as age, family history, genetic predisposition, head trauma, cardiovascular disorders, diabetes mellitus, and tobacco use (1, 29). A study that analyzed death certificate data from 1999 to 2018 in the United States has compared the regions’ mortality rates from Alzheimer’s disease and related dementias at state and county levels (30). Their finding indicated that regional factors of sex, race/ethnicity, place of death, inter-region, and disease group engender the disparity in dementia mortality. Another study that examined German health insurance data between 2014 and 2019 has found that factors such as socioeconomic status, air pollution, and healthcare access affect the risk of dementia among older adults (31). The study also explored whether these factors impacted men and women differently. Critical risk factors identified in our study included the number of persons over 60 years of age, GDP *per capita*, number of hospital beds, number of doctors, and counts of PM2.5. Previous studies on socioeconomic factors’ effects on dementia have reported that residents in circumstances with better economics and health resources lead positive, healthy lifestyles and stimulating behaviors (32, 33). While rural areas could be correlated with poor conditions and economics, and insufficient local health resources. One study used data from a cohort design of the 10/66 Dementia Study Group in middle-income countries and found that the absolute and relative risk of dementia mortality was higher in rural areas of China than in urban areas, with an estimated median standardized mortality ratio for dementia of 2.77 (6). Similarly, our model results indicated a benefit for older adults living in better situations that were helpful to get medical services and live well, resulting in a reduced risk of dementia mortality.

Our results also showed that PM2.5 exposure was positively associated with the spatial disparity in dementia mortality. Numerous studies examined the association between PM2.5 exposure and dementia mortality risk (34, 35). A 10-year study of the US veterans cohort suggests that excess deaths due to dementia are associated with PM2.5 concentrations and that the attributable burden of deaths due to dementia is exceptionally high among socioeconomically disadvantaged groups in the US (36). A meta-analysis of associations between PM2.5 exposure and neurological disorders, which included 80 studies, showed that PM2.5 exposure was strongly associated with an increased risk of dementia, AD, and Parkinson’s disease (37). The harmful effects of air pollution exposure on cognitive function, and the risk of cognitive decline, were also validated. The potential toxicological mechanism by which PM2.5 exposure induces the aggravation of dementia is neuronal damage caused by PM2.5 through causing oxidative stress, neuroinflammation, or altered dopamine levels (38, 39).

Epidemiological reports on the spatial disparity in dementia mortality in middle-income countries were limited. A systematic analysis estimates that the median standardized mortality ratio for dementia in China was 1.94 (40). Many developed countries have analyzed dementia mortality using population-based surveillance data or registration data. Using the US national death certificate data from 2000 and 2010, Xu et al. used a time-scan statistical approach to obtain the spatial and temporal clusters of dementia mortality in the north-eastern US, with a lower relative risk and the most likely temporal cluster of excess mortality located in the Pacific Northwest (41). The US Centers for Disease Control and Prevention report on dementia mortality from 2000 to 2017 shows that the age-adjusted mortality rate for dementia in 2017 was 66.7 deaths per 100,000 US standard population, with a higher age-adjusted mortality rate for women (72.7) than for men (56.4) (42). Our study is consistent with other studies reporting geographic disparities in dementia mortality. These studies can provide policymakers with relevant information to allocate scarce health resources efficiently and equitably.

There is a general lack of awareness of the disease burden of dementia in China, as many people still consider dementia as normal aging and do not have access to dementia health services for old age and care, which places an even heavier burden on the families and communities of those affected. Although dementia primarily affects older people, it is not a normal part of aging (43). In the context of severe aging, the number of counties and districts at high risk of dementia mortality in Zhejiang province has increased accordingly. One of the essential aims of this study is to note high-risk districts for dementia interventions amidst limited public health resources. This is crucial because the residential location could be a marker for healthcare services’ social and environmental factors. Spatial modeling and mapping provide the tools to obtain residents’ health outcomes. Understanding this variation and its attributable factors will contribute to disease prevention, monitoring access to health services, and planning targeted public health interventions.

It should be noted however that the geographical variations of standardized mortality ratios for four dementia subtypes are not identical. Researchers usually fit a Poisson log-linear model to estimate small spaces’ relative risk. When the observed data are scarce, a maximum likelihood approach to the above model may lead to unstable and largely uninformative maximum likelihood estimates of the area-specific linear trends due to Poisson sampling variation (23). The death data of a dementia subtype in a small area is 0 in many places in a year. Therefore, we have added up the number of deaths in 5 years of a dementia subtype and used the average population in 5 years of each county or district as a standard to calculate the standardized mortality rate to observe the geographical variations of the four dementia subtypes’ mortality. Our findings indicated that the standardized mortality ratio for vascular dementia is exceptionally high in several counties in northern Zhejiang Province, while that of unspecified dementia is particularly high in western Zhejiang. To ascertain the association between regional mortality hazards and these elevated death rates, appropriate analytical methods need to be employed.

The method conducted in this study, the Besag-York-Mollié model, is a popular Bayesian hierarchical spatial model that uses point data to exploit the correlation between nearby data points and to produce disease risk estimates in a continuous surface. The BMY model yields high-resolution disease maps that enable more accurate implementation of public health programs for optimal impact. A potential drawback of the BYM model is its assumption of a constant relative risk across all areas, which may not reflect reality in some situations. Moreover, the model relies on a predefined adjacency matrix to specify the spatial structure of the data, which may not capture all aspects of spatial dependence (22). We also conducted statistical tests on the time effect in mortality for all dementia deaths and found no statistically significant difference between years (*p* > 0.05). The mortality of four dementia subtypes remained stable overall, and a possible explanation is the stability of the population size and structure in this location.

The mortality case data for this study were obtained from health system records and may underestimate the valid mortality rate for dementia. Despite improved access to health services, inadequate diagnosis and management of dementia are still common. This may produce nondifferential or differential misclassification bias due to the lack of diagnostic capacity for dementia in community hospitals, particularly in rural areas. Due to the lack of individual disease status in the mortality data, this study did not account for other comorbidities in the analysis. This may overlook the spatial variation of chronic diseases, the health condition, and the number of comorbidities of the local population and their influence on dementia mortality across different regions. This study used current-year air pollution indicators as a proxy measure for short-term air pollution exposure and did not research the effect of longer-term air pollution exposure on dementia mortality. Furthermore, the pollution indicators in our model only included PM2.5 exposure, which is not sufficiently diverse and could have been analyzed for associations with other air pollution indicators such as sulfur dioxide (SO_2_), carbon monoxide (CO), and nitrogen dioxide (NO_2_). Limited by the data sources, this study included fewer regional factors of physical and social variables, and future studies could be improved in terms of design, analysis, and reporting to provide a solid basis for recommendations and possible interventions.

## 5. Conclusion

Our modeling reveals the spatial variations of dementia mortality risk in Zhejiang province from 2015 to 2019. We mapped dementia mortality and predicted risk, and explored the association between socioeconomic and environmental factors and dementia mortality risk as a means to prevent and control excess deaths due to dementia. Higher risks of dementia mortality were found in counties and districts with poorer economic status, insufficient health resources, and worse pollution. We suggest that appropriate measures be implemented to address the critical risk factors identified in this study, to reduce spatial disparity in dementia mortality risk. Our research has some limitations, such as underestimating the mortality rate, lack of diagnostic capacity, and unaccounted comorbidities, and it is hoped that future research will address these domains. Our work can provide some different ideas for dementia mortality analysis studies.

## Data availability statement

The original contributions presented in the study are included in the article/supplementary material, further inquiries can be directed to the corresponding authors.

## Author contributions

XH, XL, NL, and JL study concept and design. XH, XL, and XZ: data analysis. XH and XL interpretation of data. XH, XL, and NL drafting of the manuscript. XL and JL revision of the draft. All the authors read and approved the final manuscript.

## Funding

This work was granted by a Project of Zhejiang Provincial Federation of Social Sciences (2022B64), a Project of Science Technology Department of Zhejiang Province (2023C35007), the National Natural Science Foundation of China (72004201 & 71734005), and the Natural Science Foundation of Zhejiang Province (D19G030003).

## Conflict of interest

The authors declare that the research was conducted in the absence of any commercial or financial relationships that could be construed as a potential conflict of interest.

## Publisher’s note

All claims expressed in this article are solely those of the authors and do not necessarily represent those of their affiliated organizations, or those of the publisher, the editors and the reviewers. Any product that may be evaluated in this article, or claim that may be made by its manufacturer, is not guaranteed or endorsed by the publisher.
